# Combined Analysis of Phase III Trials Evaluating [^99m^Tc]Tilmanocept and Vital Blue Dye for Identification of Sentinel Lymph Nodes in Clinically Node-Negative Cutaneous Melanoma

**DOI:** 10.1245/s10434-012-2612-z

**Published:** 2012-10-03

**Authors:** Vernon K. Sondak, Dennis W. King, Jonathan S. Zager, Schlomo Schneebaum, Julian Kim, Stanley P. L. Leong, Mark B. Faries, Bruce J. Averbook, Steve R. Martinez, Christopher A. Puleo, Jane L. Messina, Lori Christman, Anne M. Wallace

**Affiliations:** 1 H. Lee Moffitt Cancer Center, Tampa, FL USA; 2 STATKING Clinical Services, Fairfield, OH USA; 3 Sourasky Medical Center, Tel Aviv, Israel; 4 University Hospitals Seidman Cancer Center, Cleveland, OH USA; 5 California Pacific Medical Center, San Francisco, CA USA; 6 John Wayne Cancer Institute, Santa Monica, CA USA; 7 MetroHealth Medical Center/Case Western Reserve University, Cleveland, OH USA; 8 UC Davis Cancer Center, Sacramento, CA USA; 9 San Diego Moores Center Institute, University of California, La Jolla, CA USA

## Abstract

**Background:**

[^99m^Tc]Tilmanocept is a CD206 receptor-targeted radiopharmaceutical designed for sentinel lymph node (SLN) identification. Two nearly identical nonrandomized phase III trials compared [^99m^Tc]tilmanocept to vital blue dye.

**Methods:**

Patients received [^99m^Tc]tilmanocept and blue dye. SLNs identified intraoperatively as radioactive and/or blue were excised and histologically examined. The primary end point, concordance, was the proportion of blue nodes detected by [^99m^Tc]tilmanocept; 90 % concordance was the prespecified minimum concordance level. Reverse concordance, the proportion of radioactive nodes detected by blue dye, was also calculated. The prospective statistical plan combined the data from both trials.

**Results:**

Fifteen centers contributed 154 melanoma patients who were injected with both agents and were intraoperatively evaluated. Intraoperatively, 232 of 235 blue nodes were detected by [^99m^Tc]tilmanocept, for 98.7 % concordance (*p* < 0.001). [^99m^Tc]Tilmanocept detected 364 nodes, for 63.7 % reverse concordance (232 of 364 nodes). [^99m^Tc]Tilmanocept detected at least one node in more patients (*n* = 150) than blue dye (*n* = 138, *p* = 0.002). In 135 of 138 patients with at least one blue node, all blue nodes were radioactive. Melanoma was identified in the SLNs of 22.1 % of patients; all 45 melanoma-positive SLNs were detected by [^99m^Tc]tilmanocept, whereas blue dye detected only 36 (80 %) of 45 (*p* = 0.004). No positive SLNs were detected exclusively by blue dye. Four of 34 node-positive patients were identified only by [^99m^Tc]tilmanocept, so 4 (2.6 %) of 154 patients were correctly staged only by [^99m^Tc]tilmanocept. No serious adverse events were attributed to [^99m^Tc]tilmanocept.

**Conclusions:**

[^99m^Tc]Tilmanocept met the prespecified concordance primary end point, identifying 98.7 % of blue nodes. It identified more SLNs in more patients, and identified more melanoma-containing nodes than blue dye.

The introduction of sentinel lymph node (SLN) biopsy revolutionized the surgical management of cutaneous melanoma, providing a minimally invasive means to stage clinically negative regional nodes and identify patients with microscopic nodal involvement for early therapeutic intervention.^1^,^2^ Despite its widespread use, the technique of performing SLN biopsy remains surprisingly nonstandardized. The original standard for defining a first-echelon or “sentinel” node draining a primary cutaneous melanoma was the visual identification of blue color in the node after injection of a vital blue dye (initially isosulfan blue or Patent Blue V) at the primary tumor site.^3^ The limited availability of these vital blue dyes led some to adopt methylene blue dye for lymphatic mapping, despite conflicting data on its comparability and safety.^4^,^5^ Most surgeons also adopted radiolabeled colloid to detect the location of SLNs preoperatively via lymphoscintigraphy and intraoperatively using a hand-held gamma radiation detecting probe in conjunction with blue dye visualization of the SLN. Several different colloids with varying properties are used around the world, with different methods of preparation (such as filtration to limit the size of the colloidal particles injected).^6^–^9^ A number of surgeons do not use blue dye at all for melanoma lymphatic mapping, preferring to rely exclusively on the radiolabeled colloid.^10^

The ideal radiopharmaceutical for melanoma lymphatic mapping would have several favorable properties: it would be standardized and require minimal preparation or modification before injection; it would be nontoxic and relatively painless, eliminating the need to infiltrate or include local anesthetics that could influence the rate of lymphatic uptake; it would be promptly taken up by the lymphatics and quickly transported to the first-echelon nodes; it would accumulate in the first-echelon nodes in high amounts, providing good contrast to background counts in the nodal basin and to the residual counts at the primary site (i.e., minimizing shine-through and maximizing step-down); and it would reside in the first-echelon nodes with minimal pass-through to second-echelon (nonsentinel) nodes. Obviously, to the degree that vital blue dyes are considered the gold standard for SLN identification, this ideal radiotracer should colocalize in those nodes that were blue stained. The ultimate goal of SLN biopsy, however, is to effectively identify tumor-containing nodes; an ideal radiotracer should identify a very high percentage of pathology-positive nodes, whether stained blue or not.

To date, no available radiolabeled colloid meets the above criteria of the ideal radiopharmaceutical SLN mapping agent, and indeed in the United States there is no radiopharmaceutical currently approved by the US Food and Drug Administration (FDA) specifically for the purpose of SLN identification in melanoma. Recently, the US FDA granted approval to technetium (^99m^Tc) sulfur colloid injection (SCI; Pharmalucence, Billerica, MA) for SLN identification in breast cancer, based entirely on retrospective data showing a high degree of concordance between nodes stained blue by blue dye and those identified as radioactive above baseline (i.e., “hot”) with SCI, along with a high rate of identification of tumor-positive nodes as hot.^11^ Such retrospective data are clearly limited by varying definitions of the threshold for considering a node to be hot, as well as the many differences in dose, formulation, and preparation inherent when SCI is used at different institutions. These limitations notwithstanding, the US FDA is expected to consider whether to approve SCI for SLN identification in cutaneous melanoma based on similar methodology in the near future.

[^99m^Tc]Tilmanocept (Fig. 1) is an engineered radiopharmaceutical specifically designed for lymphoscintigraphic and intraoperative SLN detection, created with the goal of attaining as many of the properties of the ideal radiotracer as possible.^12^ Tilmanocept is a fully standardized reagent requiring little or no manipulation before injection. Its multiple diethylene-triamine-pentaacetic acid (DTPA) moieties chelate and bind tightly to ^99m^Tc, while its multiple mannose moieties serve as ligands for multivalent binding to mannose receptors (CD206) expressed on the surfaces of reticuloendothelial cells resident in lymph nodes.^13^,^14^ Preclinical studies and phases I and II clinical trials have provided evidence that [^99m^Tc]tilmanocept fulfills many of the desired characteristics of the ideal radiotracer for SLN identification.^12^,^15^–^19^ Its chemical structure and relatively small size (molecular weight ≈ 19,000 Da, diameter 7.1 nm) enable [^99m^Tc]tilmanocept to exit the injection site rapidly and quickly accumulate in first-echelon nodes. Within lymph nodes, interactions between [^99m^Tc]tilmanocept’s mannose moieties and CD206 enable avid binding to the target receptor and retention for up to 30 h (Fig. 2), potentially limiting transit to second-echelon nodes.^20^ In phase I trials involving small numbers of melanoma and breast cancer patients, [^99m^Tc]tilmanocept showed favorable properties when directly compared to patients injected with sulfur colloid in the same study.^16^,^17^ In a phase II trial, 49 melanoma and 31 breast cancer patients underwent SLN biopsy using [^99m^Tc]tilmanocept with or without blue dye.^19^ At least one SLN was identified by [^99m^Tc]tilmanocept in 96.2 % of 78 evaluable patients, including 46 of the melanoma patients, with an average of 2.3 hot nodes identified per melanoma patient. Eighteen of 19 nodes found to contain melanoma and 8 of 9 nodes with breast cancer were identified by [^99m^Tc]tilmanocept. No patients experienced adverse events the investigators considered probably or definitely related to [^99m^Tc]tilmanocept.^19^

**Fig. 1:**
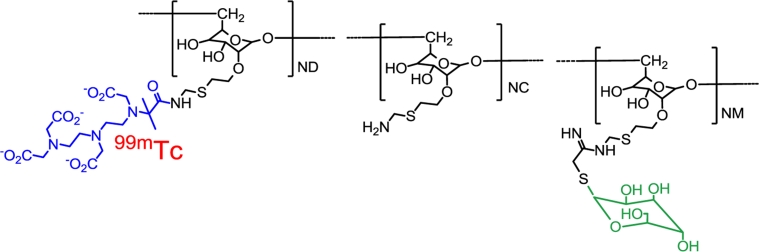
Chemical structure of [^99m^Tc]tilmanocept. [^99m^Tc]Tilmanocept is composed of a dextran backbone (*black*) to which are attached multiple units of mannose (*green*) and DTPA (*blue*). The mannose units provide a molecular mechanism by which [^99m^Tc]tilmanocept avidly binds to a receptor specific to reticuloendothelial cells (CD206), and the DTPA units provide a highly stable means to radiolabel tilmanocept with ^99m^technetium (*red*). The molecular weight of [^99m^Tc]tilmanocept is approximately 19,000 g/mol; the molecular diameter is 7.1 nm (adapted from Wallace et al.^16^)

**Fig. 2:**
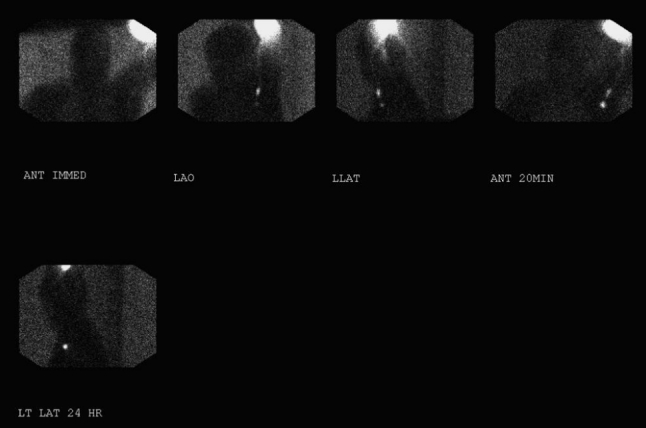
Preoperative lymphoscintigraphy with [^99m^Tc]tilmanocept. Images were obtained over 24 h in a 72-year-old melanoma patient undergoing SLN biopsy for a 0.95-mm nonulcerated melanoma. The patient received four intradermal injections, each consisting of 0.49 mCi of [^99m^Tc]tilmanocept, at the primary site on the left forearm (*first image*, *top row*) and one axillary SLN was visualized at 20 min (*second through fourth images*, *top row*), along with activity in lymphatic channels of the upper arm seen superior and distal to the SLN. The same SLN visualized at 20 min was observed at 24 h (*bottom row*). Intraoperatively, approximately 1 h after the final image was obtained, the tissue background 10-second count was 50 (3σ = 71, see text) and a single blue axillary node with 10-s counts of 1,521 was found corresponding to the SLN on the images. This node had no histologic evidence of melanoma

An ultimate assessment of the suitability of [^99m^Tc]tilmanocept for use in melanoma SLN identification, however, requires larger patient numbers in a phase III trial setting. This report describes results of two phase III trials with basically identical designs: open-label, nonrandomized trials in which clinically node-negative patients received both [^99m^Tc]tilmanocept and blue dye for SLN identification. The goal of these trials, which included both melanoma and breast cancer patients, was to evaluate the concordance (colocalization within the same lymph node) of [^99m^Tc]tilmanocept and vital blue dye, while also determining the safety profile of [^99m^Tc]tilmanocept and how often [^99m^Tc]tilmanocept identified tumor-containing nodes that were not blue-stained. This trial design was not intended to compare [^99m^Tc]tilmanocept to any other radiocolloid. We describe the summed results for melanoma patients enrolled onto these two trials.

## Methods

### Study Design

Two phase III trials were performed on patients without preoperative clinical evidence of nodal metastases undergoing clinically indicated SLN biopsy for cutaneous melanoma. Evaluating concordance of [^99m^Tc]tilmanocept with blue dye was the primary end point of both studies. Both were prospective, open-label, nonrandomized, within-patient trials in which all patients were to receive both [^99m^Tc]tilmanocept and a vital blue dye (isosulfan blue [Lymphazurin] or Patent Blue V). Nine sites enrolled patients June 2008–June 2009 in the first trial, while six sites enrolled patients July 2010–April 2011 in the second. Other than the sites involved and the number of patients per site allowed, the two studies were virtually identical. This report describes results obtained from the melanoma patients who participated in these two trials.

Enrollment criteria included the histologically confirmed presence of cutaneous melanoma ≥0.76 mm with a surgical treatment plan including SLN biopsy. Table 1 lists complete eligibility criteria. Patients received a fixed dose of 50 μg of [^99m^Tc]tilmanocept (~2.7 nmol) administered by intradermal injection. For surgery on the day of the injection, patients received ~0.5 mCi of ^99m^Tc, while next-day surgery patients received ~1.0–2.0 mCi (timing of injection was at the surgeon’s discretion). Blue dye was injected intradermally after [^99m^Tc]tilmanocept, at the time of surgery.

**Table 1:** Study enrollment criteria

Inclusion criteria
Confirmed presence of cutaneous melanoma with clinically negative nodes
Candidate for surgical intervention, with sentinel node biopsy part of the surgical plan
At least 18 years of age
ECOG performance status of 0–2
If female, either negative pregnancy test within 72 h before [^99m^Tc]tilmanocept administration or surgically sterilized or postmenopausal for at least 1 year before study entry
Exclusion criteria
Pregnancy or lactation
Clinical or radiological evidence of metastatic cancer, including palpably abnormal or enlarged lymph nodes
Known hypersensitivity to isosulfan blue or Patent Blue V
Participation in another investigational drug study
Melanoma with a Breslow depth of <0.76 mm
Prior diagnosis of an invasive melanoma occurring on the same body region or potentially draining to the same nodal basin
Truncal or extremity primary melanoma and a prior breast cancer potentially draining to the same axillary nodal basin
Preoperative chemotherapy, immunotherapy, or radiotherapy
Node basin surgery of any type or radiation to the nodal basin(s) potentially draining the primary melanoma
Prior wide excision (>1 cm in dimension) or complex reconstruction for the primary melanoma

### Criteria for Intraoperative Identification of SLNs

**Fig. 3:**
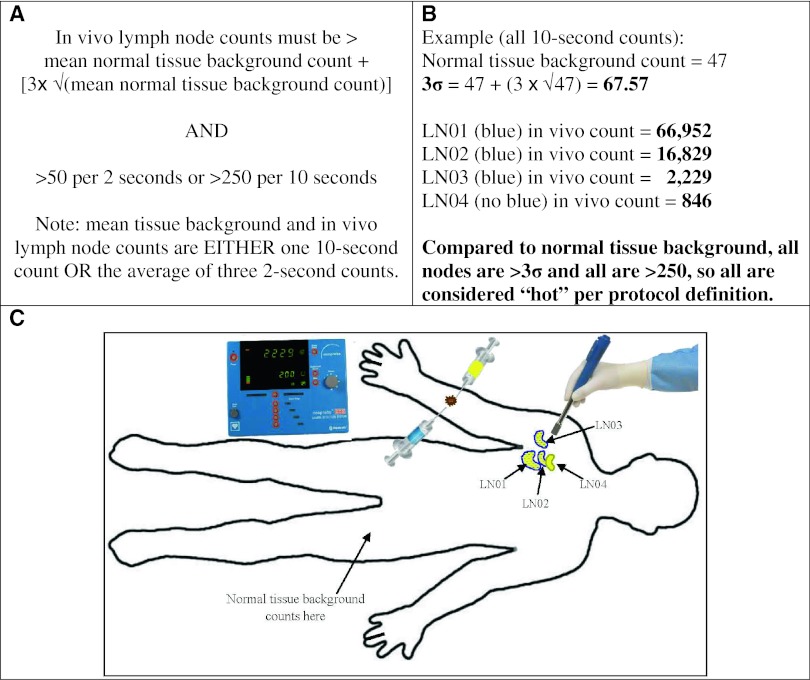
The 3σ rule. **a** The equation for determining whether the in vivo radioactive counts in a lymph node are estimated to be at least three standard deviations above the normal tissue background count. **b** Example of 3σ calculation for a protocol patient with four SLNs identified intraoperatively. All in vivo node counts were greater than the 3σ value, and all in vivo counts were >250. All were therefore considered hot per the protocol definition. **c** Schematic of injections of vital blue dye (*blue syringe*) and [^99m^Tc]tilmanocept (*gold syringe*) intradermally at the primary tumor site (*brown circle*) with lymphatic mapping with a handheld gamma detection probe. In this example, nodes 1 through 3 are shown as hot and blue (i.e., per lymph node concordance 3/3 = 100 %; per patient concordance = yes), while node 4 is only hot (i.e., reverse concordance 3/4 = 75 %)

Three criteria were utilized for intraoperative identification of SLNs. Briefly, the first criterion was in vivo visualization of blue dye in a node and/or its afferent lymphatic channel. All such nodes were designated as blue and removed. The second criterion was based on radioactivity; background counts (using either one 10-s count or the average of three 2-s counts), were measured directly on the patient ≥20 cm from the primary site and nodes were interrogated using a Neoprobe gamma detector before (in vivo) and after excision (ex vivo). To qualify as radioactive (hot), the in vivo counts in the node had to (1) exceed the background count *plus* three times the standard deviation of the background (termed the 3σ rule; corresponding to the 99.7 % confidence limits; Fig. 3) and (2) average at least 50 (for three 2-s counts) or exceed 250 (for one 10-s count). The third criterion for intraoperative identification of SLNs was any palpably enlarged or abnormal node. All excised nodes underwent histological evaluation with hematoxylin and eosin and immunohistochemistry using S-100 and HMB-45 and/or MART-1/Melan-A antibodies.

### Statistical Plan

The statistical plan defined the population for assessing concordance as all enrolled patients who received both [^99m^Tc]tilmanocept and blue dye *and* who had at least one blue-stained histologically confirmed lymph node. Drug safety was analyzed on all enrolled patients who received [^99m^Tc]tilmanocept, whether or not they received blue dye. The primary efficacy end point, the per lymph node concordance of [^99m^Tc]tilmanocept with blue dye, was defined as the number of blue-stained nodes that were detected by [^99m^Tc]tilmanocept, divided by the number of blue-stained lymph nodes. Concordance was tested in a pooled analysis with the alternative hypothesis that [^99m^Tc]tilmanocept would be considered concordant to blue dye if >90 % of blue nodes were radioactive per the above definition. A 95 % two-sided exact binomial confidence interval (CI) for concordance was calculated; a two-sided *p* value of ≤0.05 indicated that the null hypothesis was rejected. A secondary end point was the per patient concordance rate, defined as the percentage of patients for whom all nodes detected by blue dye were detected by [^99m^Tc]tilmanocept. Another secondary end point, reverse concordance, evaluated the proportion of all nodes detected by [^99m^Tc]tilmanocept that were also blue. The proportions of pathologically positive lymph nodes that were blue and/or radioactive were compared using McNemar’s exact test.

### Study Oversight and Role of the Sponsor

The protocols, patient instructions, and informed consent documentation were approved by the US FDA, the Western Institutional Review Board, and the institutional review boards of each enrolling institution. These studies complied with all provisions of the Declaration of Helsinki and US laws requiring registration and updates via ClinicalTrials.gov (trial numbers NCT00671918 and NCT01106040). All participants gave voluntary written informed consent. The trial was designed by the sponsor, Navidea Biopharmaceuticals, who supplied tilmanocept cold kits for radiolabeling to each study site. Data were analyzed independently by STATKING Clinical Services in collaboration with the authors, who vouch for the completeness and accuracy of the data and analyses. An initial draft of this article was prepared by the first author and reviewed by Navidea medical personnel, independent statistical reviewers, and auditors. All authors contributed to subsequent drafts and approved the final document for submission.

## Results

### Patient Population

A total of 170 melanoma patients were enrolled in the two phase III trials, of whom 14 were not injected with [^99m^Tc]tilmanocept because of withdrawal of consent or trial conclusion before scheduled surgery. The demographic characteristics of the 156 enrolled patients with melanoma who satisfied the inclusion and exclusion criteria and were injected with [^99m^Tc]tilmanocept are given in Table 2. There was a predominance of male patients (61.5 %), and most patients (68.6 %) had intermediate-thickness melanomas (1.01–4.00 mm in thickness, stage T2 or T3). Two patients were injected with [^99m^Tc]tilmanocept but were not injected with blue dye; these patients were evaluable for safety of [^99m^Tc]tilmanocept but not for concordance.

**Table 2:** Demographics of 156 patients receiving [^99m^Tc]tilmanocept

Characteristic	Value
Sex, *n*	
Male	96
Female	60
Age (years)	
Median	60.0
Range	20–90
Weight (lb)	
Median	190.0
Range	96.9–372.6
Race, *n* (%)	
White	154 (98.7 %)
African American	2 (1.3 %)
Asian	0
Other	0
Ethnicity, *n* (%)	
Non-Hispanic	151 (96.8 %)
Hispanic	1 (0.6 %)
Unknown	4 (2.6 %)
Tumor stage, *n* (%)	
T1	29 (18.6 %)
T2	72 (46.2 %)
T3	35 (22.4 %)
T4	20 (12.8 %)

Includes two patients who never received vital blue dye and who are excluded from concordance analyses

### Intraoperative Node Identification

**Fig. 4:**
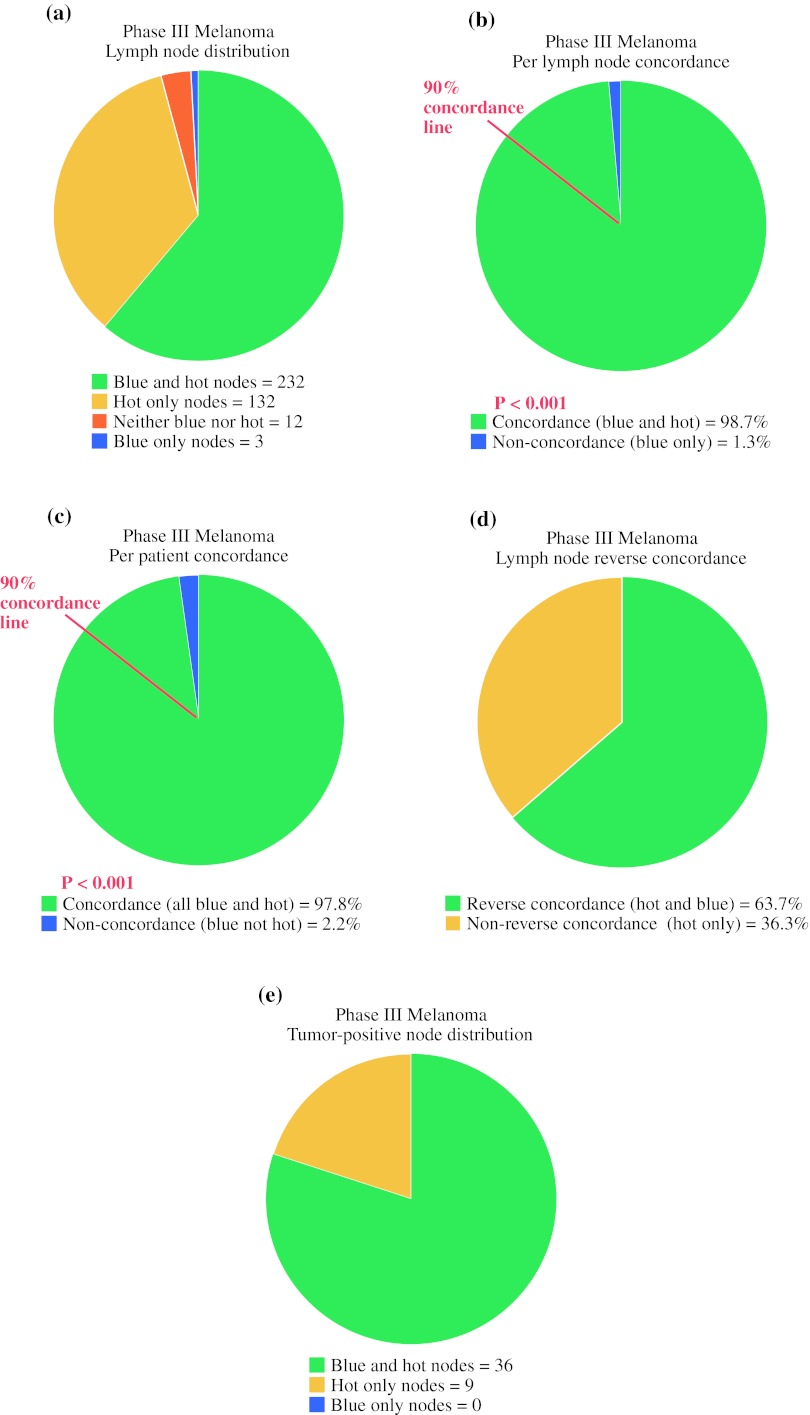
Distribution of excised lymph nodes and concordance of [^99m^Tc]tilmanocept with vital blue dye. **a** Lymph node distribution, based on a total of 379 excised lymph nodes. Twelve lymph nodes removed from nine patients were not blue and did not meet the protocol definition for radioactivity above background; none contained melanoma. **b** Per lymph node concordance, based on a total of 235 blue lymph nodes. The statistical threshold for concordance was prospectively set as 90 % of blue nodes would be hot. (In **b** and **c**, the *red line* marks the 90 % concordance threshold.) **c** Per patient concordance, based on 138 patients with at least one blue lymph node. **d** Per lymph node reverse concordance, based on a total of 364 hot nodes. Only 232 (63.7 %) were also blue. **e** Tumor-positive lymph node distribution, based on 45 lymph nodes found to contain melanoma by routine histology and/or immunohistochemistry. No tumor-positive nodes were blue but not hot

In all, 154 patients injected with both [^99m^Tc]tilmanocept and blue dye underwent surgery for evaluation of their sentinel nodes, of whom 138 patients (89.6 %) had at least one blue node and 150 patients (97.4 %) had at least one radioactive node. The observation that [^99m^Tc]tilmanocept detected a node in more patients than blue dye was statistically significant (*p* = 0.002). There were 379 lymph nodes excised; 235 were blue and 364 were radioactive (Fig. 4a). The 138 patients with at least one blue node had a mean of 1.7 blue nodes detected per patient. The 150 patients with at least one radioactive node had a mean of 2.4 radioactive nodes detected per patient. Three patients did not have a lymph node identified intraoperatively by either [^99m^Tc]tilmanocept or blue dye: one patient exhibited multiple subclavian and retrosternal hot spots upon lymphoscintigraphy, but as a result of their location, the investigator elected not to excise the nodes, and two patients had two nodes removed that were neither hot per the protocol definition nor blue.

### Concordance of [^99m^Tc]Tilmanocept and Vital Blue Dye

The primary study end point was the concordance between lymph nodes identified as radioactive with [^99m^Tc]tilmanocept and lymph nodes identified as blue with vital blue dye. There were 235 blue lymph nodes identified; of these, 232 (98.7 %, 95 % CI 96.3–99.7) were also radioactive (Fig. 4b). This rate of per lymph node concordance was statistically significant (*p* < 0.001), convincingly rejecting the null hypothesis of ≤90 % concordance. For the 138 patients with at least one blue node, 135 patients had all of their blue nodes also radioactive, for a per patient concordance rate of 97.8 % (95 % CI 93.8–99.5, *p* < 0.001, Fig. 4c).

Reverse concordance was defined as the percentage of radioactive lymph nodes that were also blue. There were 364 radioactive lymph nodes identified; of these, only 232 (63.7 %, 95 % CI 58.6–68.7) were also blue (Fig. 4d). Similarly, in only 76 of 150 patients with at least one radioactive node (50.7 %) were all the radioactive nodes also blue.

### Pathologic Findings

Among the 154 melanoma patients who were injected with both [^99m^Tc]tilmanocept and blue dye, there were a total of 45 tumor-containing nodes in 34 patients (22.1 % SLN positivity rate). [^99m^Tc]Tilmanocept detected all of these tumor-positive lymph nodes (Fig. 4e). Conversely, blue dye identified only 36 of the 45 positive nodes (80 %), missing an involved node in nine patients (26.5 % of node-positive patients). The difference between the rates of tumor-positive radioactive and blue lymph nodes was statistically significant (*p* = 0.004). Thus, [^99m^Tc]tilmanocept exhibited a higher detection rate for melanoma-containing SLNs than blue dye. Overall, 4 of 154 patients (2.6 %) were staged correctly only by [^99m^Tc]tilmanocept; no patients were upstaged by blue dye findings alone.

### Adverse Events

There were no patient deaths reported on either trial. There were no immediate or delayed hypersensitivity reactions to [^99m^Tc]tilmanocept. A total of 45 adverse events occurred in 29 of the 156 melanoma patients receiving [^99m^Tc]tilmanocept. Ten serious adverse events were reported from nine patients, none of which were deemed by the investigator as probably or definitely related to [^99m^Tc]tilmanocept (two cases of cellulitis, and one case of each of nausea, vomiting, seroma, syncope, asthma, bradycardia, tachycardia and myocardial infarction). The most common adverse events of any severity were cellulitis (seven patients), incision site pain (four patients), nausea (three patients), and seroma (three patients); most adverse events were mild or moderate in severity.

## Discussion

Sentinel node biopsy is widely accepted worldwide as an important part of the management of clinically localized cutaneous melanoma.^2^,^21^ Despite this worldwide acceptance, there is surprisingly little standardization in the technique, with a variety of different radiocolloids and vital blue dyes being used. Although isosulfan blue and Patent Blue V dyes remain the gold standard vital dyes for identifying SLNs, not all surgeons use them.^4^,^10^ Radiocolloids are almost universally used in melanoma, but there is no agreement about which colloid to use and whether to filter it before use to restrict the particle size. The inescapable conclusion is that the ideal radiopharmaceutical for melanoma lymphatic mapping has not yet been established.

[^99m^Tc]Tilmanocept was developed specifically for SLN identification and has shown favorable results in phase I and II testing in melanoma and breast cancer.^12^,^16^–^20^ These results led to the conduct of two phase III nonrandomized trials evaluating [^99m^Tc]tilmanocept in patients with melanoma or breast cancer undergoing clinically indicated SLN biopsy with either isosulfan blue or Patent Blue V dye. The primary end point of these trials was to establish the degree of concordance between blue dye and [^99m^Tc]tilmanocept; specifically, the trials were powered to determine whether more than 90 % of blue nodes were also radioactive. In 235 blue lymph nodes from 154 melanoma patients, 232 (98.7 %) were radioactive per the protocol definition. This result established with a high degree of statistical probability that [^99m^Tc]tilmanocept is in fact concordant with vital blue dye above the prespecified 90 % concordance level. Similar results were seen for patients with breast cancer; these results will be published separately.

In addition to meeting the prespecified primary efficacy end point, these two phase III trials established that [^99m^Tc]tilmanocept plus blue dye identified at least one SLN in a very high percentage of melanoma patients (97.6 %), that [^99m^Tc]tilmanocept identified more nodes per patient than were visibly stained blue, and most importantly that a substantial fraction of melanoma-containing lymph nodes were radioactive but not visibly blue-stained (20 % of all positive SLNs). [^99m^Tc]Tilmanocept had a favorable safety profile, with no serious adverse events ascribed to the agent, and injections were well tolerated without any accompanying local anesthetics. Although not specifically assessed as part of the trial design, study investigators (all experienced melanoma surgeons) perceived the [^99m^Tc]tilmanocept lymphoscintigrams to be of high quality.

These phase III trials do not, however, allow for a direct comparison between [^99m^Tc]tilmanocept and other available radiopharmaceuticals for melanoma lymphatic mapping. Potential advantages of its small molecular size and the receptor-targeted nature of the mannose moieties in [^99m^Tc]tilmanocept include rapid transit from the primary site to the SLN and selective accumulation in that node, with limited pass-through to second-echelon nodes. A radiopharmaceutical possessing these properties might be particularly advantageous when the primary melanoma is in close proximity to the sentinel node and/or for use in conjunction with single positron emission computed tomography (SPECT) imaging.^22^,^23^ In this regard, Fig. 2 shows a patient wherein the primary SLN is distinguished within 20 min of the injection, and this SLN retained [^99m^Tc]tilmanocept 24 h later. Thus, this agent could be particularly well suited for use in conjunction with SPECT imaging and merits further evaluation in this setting.

The rigorous data obtained in these two phase III trials strongly support [^99m^Tc]tilmanocept as a safe and effective agent for lymphatic mapping and intraoperative identification of melanoma SLNs.
